# Swyer-James Syndrome: A Rare Radiological Report of Two Cases Presenting in Adulthood

**DOI:** 10.7759/cureus.63123

**Published:** 2024-06-25

**Authors:** Khavin Kumar, Jeffrey S Joseph, Ramprasath Sathiamoorthy, Rajamani Anand, Einstien Arulraj

**Affiliations:** 1 Department of Radiology, Chettinad Hospital and Research Institute, Chettinad Academy of Research and Education, Chennai, IND

**Keywords:** hyperlucency of lungs, bilateral mosaic pattern, chest imaging of sjs, macleod syndrome, adult swyer-james syndrome

## Abstract

Swyer-James syndrome (SJS), also termed MacLeod syndrome, is an acquired secondary unilateral hyperlucency of the lung due to childhood lung infections. This disorder can be diagnosed in children; however if there are few or no symptoms, diagnosis can be missed and can then be detected later in adult life as an incidental finding. We present here the case reports of two patients, where one of them had a unique presentation of unilateral hyperlucency on a chest radiograph and a bilateral mosaic pattern on CT lung but with no history of childhood infections and another case with unilateral hyperlucency of the lung with the history of childhood infection were diagnosed as SJS. This article is important as it highlights the significant radiological finding in accurately diagnosing this condition, when the presenting complaint and past history are inconclusive, thereby guiding proper management.

## Introduction

Swyer-James syndrome (SJS) is an acquired childhood condition which is otherwise termed MacLeod syndrome. This condition is characterized by unilateral hyperlucency of the lung which is most commonly secondary to infectious bronchiolitis that may lead to lung growth retardation, hyperinflation due to obstruction, and hypoplasia of pulmonary vessels [1]. It is a long-term complication due to an acute attack of viral bronchiolitis in infants and childhood period, like adenovirus and measles which can be single or recurrent attacks [2,3]. This syndrome can be diagnosed in childhood but sometimes remains missed or underdiagnosed clinically due to fewer or no symptoms [4]. Patients with this syndrome present with symptoms similar to asthma, emphysema, and chronic obstructive pulmonary disease (COPD), so ruling out these differentials is necessary [1,3]. Here, the case reports and treatment journeys of two patients who were diagnosed in adulthood with SJS in our hospital are discussed.

## Case presentation

Case 1 

**Figure 1:**
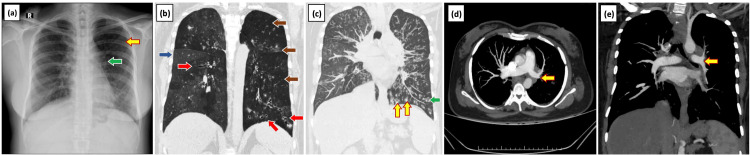
Swyer-James syndrome diagnosed in a 39-year-old female with a history of recurrent respiratory tract infection in childhood (a) Chest radiograph shows unilateral hyperlucency of the left hemi thorax (yellow arrow) with small left hilum (green arrow), reduced vascular markings in the left hemithorax, and apparent small left descending pulmonary artery. CT Coronal MinIP (b) and coronal MIP (c) reconstructed images of the chest show mosaic attenuation (blue arrow) in the right lung and bilateral bronchiectasis (red arrow) with associated mucus plugging (yellow arrow) more pronounced in the left lung. There are diffuse hypoattenuation (brown arrow) and a relatively small volume of the left lung. Small centrilobular nodules (green arrow) in both lungs suggest an active infection. Axial section CT pulmonary angiogram (d) and reconstructed coronal MIP images (e) show decreased caliber of the left main pulmonary artery (yellow arrow) and its lobar and segmental branches. MIP: Maximum intensity projection

A 39-year-old female, with a known case of type 2 diabetes mellitus, with a significant history of recurrent hospital admissions for productive cough since childhood, presented with chief complaints of dyspnea and cough with mucopurulent expectoration for the past one month. Dyspnea was progressive from Modified Medical Research Council (MMRC) grade 1 to MMRC grade 2 and sputum was neither blood-stained nor foul-smelling. She has had weight loss and loss of appetite for one month. The patient was not a known case of tuberculosis and had a negative allergic history. On examination, the patient appeared pale and afebrile with 98% saturation but tachypneic. On auscultation of breath sounds, bilateral diffuse fine inspiratory crepitation was heard which was more on the left hemithorax. A sputum sample was taken and sent for culture and broncho alveolar lavage was performed. Intravenous antibiotics and nebulization were started. Chest radiography revealed unilateral hyperlucency with reduced vascular markings on the left side for which SJS was one of the suspected differentials. On CT, the left lung was relatively smaller in volume with mild hyperinflation of the right lung and shift of the anterior junction line to the left. There were diffuse hypoattenuation and bronchiectasis in the left lung, more pronounced in the left lingula. CT pulmonary angiogram revealed decreased caliber of the left main pulmonary artery and its lobar and segmental branches. Centrilobular nodules in a tree in bud pattern were noted in bilateral lung fields predominantly on the right side indicating infective etiology (Figure 1). Sputum culture showed Klebsiella growth. We confirmed our diagnosis and provided supportive care. Amikacin and doxycycline were the antibiotics added. The patient improved symptomatically and was discharged. On follow-up, the lung imaging findings remained the same.

Case 2

**Figure 2:**
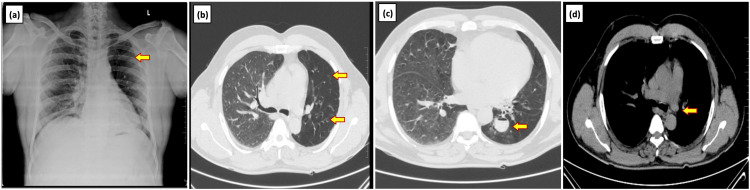
Swyer-James syndrome diagnosed in a 53-year-old male presenting with fever and productive cough (a) Chest radiograph PA view shows unilateral hyperlucency, decreased bronchovascular markings and volume loss of the left lung (yellow arrow). Axial section CT chest (lung window) (b and c) shows diffuse hypoattenuation (arrow) of the left lung and dilated bronchi with the fluid level in the superior segment of the left lower lobe [arrow]. (d) Axial CT chest (mediastinal window) shows reduced caliber of left main pulmonary artery (yellow arrow) PA: Posteroanterior

A 53-year-old male with no known comorbidities presented with chief complaints of intermittent fever, productive cough, and headache for one week. The sputum was mildly blood-stained with no foul smell. He had a history of generalized weakness and headaches associated with giddiness. There was no significant past history and he had no known exposure to tuberculosis. There was no history of chest pain, loss of weight or appetite and no addictions. On examination, the patient was afebrile and all vitals were within the normal range. On auscultation, expiratory wheeze in bilateral lower zones and crepitus in the left lower zone were heard. Chest radiograph was done which revealed unilateral hyperlucency, decreased bronchovascular markings, and volume loss of the left lung. CT findings revealed diffuse hypoattenuation, volume loss, and reduced caliber of pulmonary vessels with cystic bronchiectatic changes in the left lung, predominantly in the left lower lobe. Mucosal plugs in left secondary bronchi were also seen with few dilated bronchi showing fluid levels (Figure 2). Mild focal mosaic attenuation was noted in the medial basal segment of the right lower lobe. We came to the radiological diagnosis of SJS. Investigations like total leukocyte count, serum electrolytes, liver function test, and urine examination were done and all were normal. We prescribed tranexamic acid antibiotics, proton pump inhibitors, and antihistamines for the patient following which he recovered and as there was no further episode of hemoptysis, he was discharged. 

## Discussion

A case of SJS was first reported by Swyer and James in a child in 1953 and later by MacLeod in an adult. Since then, its occurrence and the development of this disorder have led to many controversies [5]. Although the occurrence is said to be in childhood [4,6]. Fontes et al. reported that it must be an important differential diagnosis in adulthood because it is rarely diagnosed in children or adolescents [7]. Like the patients in this study, during the literature search we found that the majority of the cases were diagnosed in adulthood [1,3,5-7]. Many authors observed that SJS had a significant history of lung infections in childhood similar to our first case [1,3,4,6,8]. However, Sen et al. [3] published in their case report that three out of four cases had no history of any childhood infections related to the lung which is similar to the second case. According to Fontes et al., childhood infections that cause bronchial epithelial injury are the main factor for triggering inflammation in the lungs, which leads to long-term destruction of alveoli and eventually submucosal fibrosis with airflow limitation and capillary bed obstruction [7]. This leads to symptoms like dyspnea, chest pain, productive cough, hemoptysis, and fever which might be chronic or recurrent [3,5,7] as seen in the first case. However, the second case having SJS presented with acute symptoms and no history of childhood infections. Both of our cases were not known to have tuberculosis, but it is essential to note that Ozcan et al. [9] and Sen et al. [3] published tuberculosis as one of the possible etiological factors for the development of SJS. Hence, in known cases of tuberculosis with no relief of symptoms, SJS needs diagnostic consideration. Like our second patient with no dyspnea, Sen et al. [3] also observed that the prominent symptom was not dyspnea because only two out of four cases had dyspnea. Few patients may present with productive cough with mucopurulent sputum or hemoptysis based on the extent of inflammation and also the type of secondary infection [3,7]. Thus sputum culture plays an important role in treating secondary infection that complicates the disorder. Fever and pleuritic thoracalgia symptoms are also present [3,7]. The clinical examination findings vary which makes clinical diagnosis difficult. Pulmonary auscultation might reveal either reduced breath sounds or wheeze or creptitations or rales or a combination [3,7]. Mostly unilateral abnormal sounds are heard but can also be heard bilaterally as noted in both our cases. Hypoxemia was not detected in any of the cases [3]. These clinical features may lead to misdiagnosis and wrong treatment.

Every reviewed article [1-10] noted that radiological findings are the gold standard for diagnosis of SJS. On chest radiography, the major finding is unilateral hyperlucency but Moore et al. [8] revealed that bilateral hyperlucency was noted in one of his eight cases and Bandeira et al. [4] observed patchy lucency in the contralateral lung. Usually, these lucencies can be diffuse but mostly limited to one lobe. It must be noted that hyperlucency also varies in inspiratory and expiratory radiographs. Hyperlucency can be poorly marginated or well demarcated as sharp-wedge-shaped [8]. Reduced bronchovascular markings, hilar markings, and volume loss of the lung are also noted [1-8]. Volume loss also led to a mediastinal shift toward the same side [4]. Computed tomography of the lung in both inspiratory and expiratory phases showed diffuse hypoattenuation of lung fields predominantly unilateral but bilateral in few cases [1-8]. Expiratory air trappings are seen in both the ipsilateral and contralateral sides of the lung which is observed as a mosaic pattern [3,4,8]. Dirweesh et al. proposed that only 30% of cases presented with bronchiectasis [10]. Unlike the above statement, our cases and other authors [3,5,8] also noticed the presence of cystic or tubular or varicoid bronchiectasis which might be limited to ipsilateral single lobe or multiple lobes or involving contralateral lung too. Pulmonary function tests reveal obstruction in the airway and ventilation-perfusion scanning shows reduced perfusion [3]. CT angiography of the lung reveals pulmonary artery asymmetry [6]. The reduced caliber of pulmonary vessels, reduced lung vasculature, hypoplasia of vessels, and reduced vessel size were also noted [1-5, 7]. The reduced caliber of vessels in the affected lung with ipsilateral pulmonary artery asymmetry was appreciated in both our cases. Although MRI angiogram is not commonly used, it is an alternative imaging modality that can demonstrate unilateral diminished pulmonary vasculature. However, the lung findings cannot be optimally assessed on MR and therefore a combination CT chest with CT angiogram is better recommended.

**Figure 3:**
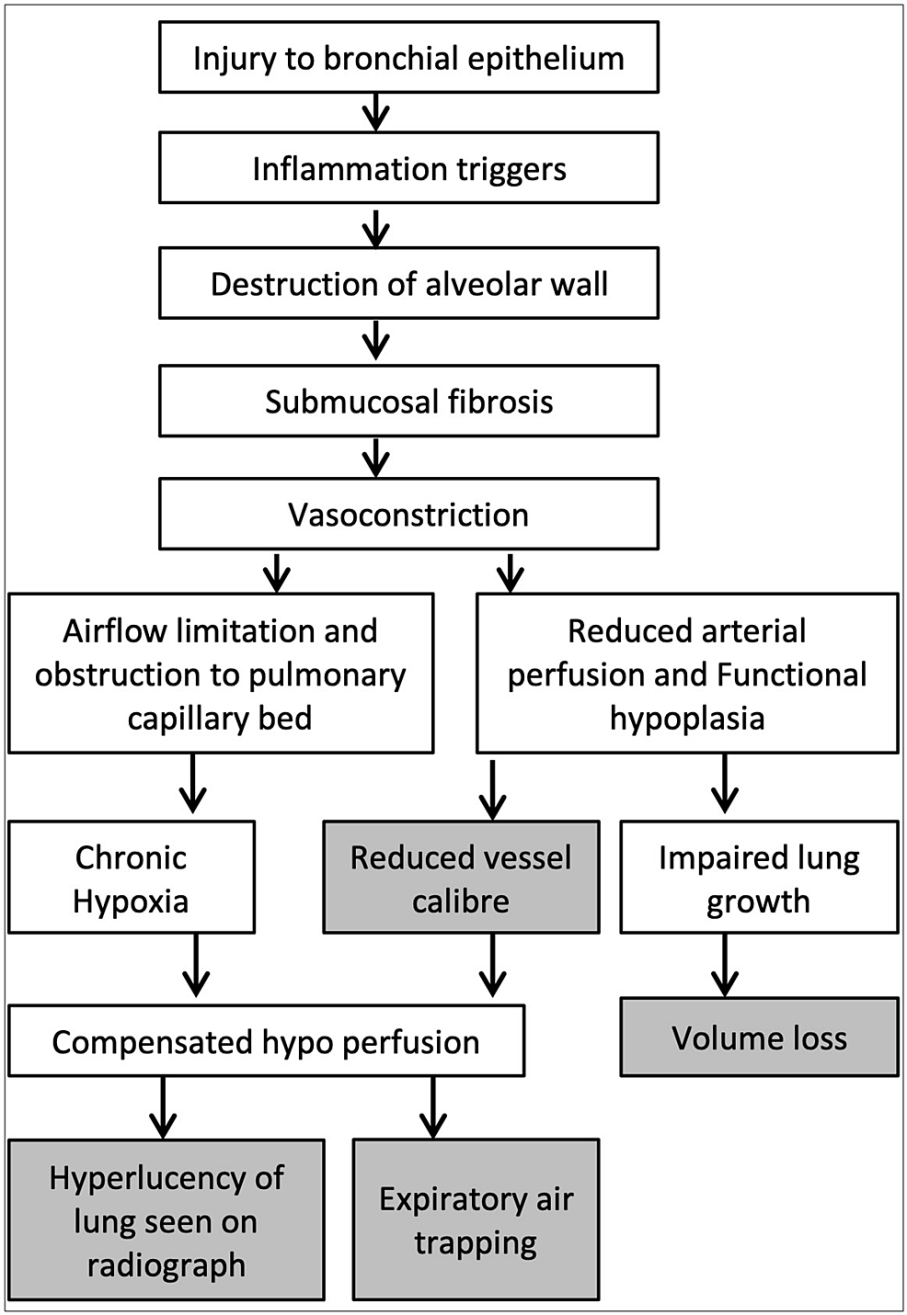
Pathogenesis of Swyer-James syndrome Original work prepared by our authors illustrating the pathophysiology of bronchial injury and its consequences.

Figure 3 presents the flow chart of pathogenesis that is derived from a few articles [6,7].

Hamada et al. mentioned that he performed parametric response mapping in these patients who demonstrated expiratory air trappings which differentiated emphysematous lesions from non-emphysematous ones [1]. He also mentioned the need for multimodal imaging diagnosis which is non-invasive [1]. Table 1 includes the differential diagnosis for such cases [4,7].

**Table 1:** Differential diagnosis of Swyer-James syndrome Source: [4,7]

Congenital	Acquired
Pulmonary artery agenesis/aplasia/hypoplasia	Swyer-James-MacLeod syndrome
Congenital lobar over inflation	Endobronchial obstruction with air trapping
Congenital lobar or interstitial emphysema	Emphysema

Other differentials for unilateral hyperlucent lung include chest wall conditions such as Poland syndrome, mastectomy, or other lung-based pathologies like pneumothorax, unilateral bulla, pulmonary embolism, and post pneumonectomy status.

In both our cases, there were no relevant prior surgical history and CT scans were efficient to rule out other causes of unilateral lung hyperlucency. In unilateral pulmonary artery atresia, there will be the absence of the main branch pulmonary artery on the affected side with the presence of collaterals; however, this was not seen in our cases.

Sputum and blood samples for culture and sensitivity must be done to know the secondary infections as the treatment modality in managing SJS syndrome is conservative [1,5,7]. Misdiagnosis as asthma or COPD will not favor the patient's outcome and might lead to over administration of steroids and further complicate the disease [3]. As the management is conservative, preventive strategies like vaccination against pneumococcus and influenza can be done. In severe cases of compensated hyperinflation, surgery for lung volume reduction can be performed [7].

## Conclusions

The importance of these case reports is that not all cases of SJS present with dyspnea having unilateral lung lucencies since bilateral involvement is also seen. History of childhood infection and chronic symptoms are not definite indicators of this disorder since asymptomatic patients may present with an acute onset of symptoms. Key imaging features such as unilateral hyperlucency, volume loss, unilateral pulmonary artery hypoplasia, and reduced caliber of vessels with or without bronchiectatic changes should indicate the likely diagnosis of SJS. Additional prior history of recurrent childhood infections with classic imaging signs should reinforce the diagnosis of SJS. Prompt diagnosis and management to prevent and cure secondary infections remain the standard due to the rare prevalence and indefinite clinical symptoms of this condition, which might lead to misdiagnosis.
